# Artificial Dilatation of the Cervix in Obstetrics1Read before the Medical and Surgical Society of Hornell. N. Y., November 2, 1908.

**Published:** 1908-12

**Authors:** James E. King

**Affiliations:** Adjunct Professor of Obstetrics, University of Buffalo, Attending Gynecologist to Buffalo General and Erie County Hospitals; Fellow of the Royal Society of London, etc.


### Artificial Dilatation of the Cervix in Obstetrics.[1]

By JAMES E. KING, M.D.

Adjunct Professor of Obstetrics, University of Buffalo, Attending Gynecologist to Buffalo General and Erie County Hospitals; Fellow of the Royal Society of London, etc.

DURING the year every general practitioner meets cases, requiring instrumental delivery. In the intrauterine application of forceps, his first concern is the state of the cervix. Is it dilated and if not is it easily dilatable? With an incompletely dilated cervix, but one which is soft and yielding, he may adopt one of two means for its dilatation. Either he may stretch the cervix manually until the dilatation is complete, or he may apply the forceps as soon as the os will admit the blades and dilate by the advancing head. This latter practice, while condemned by some authorities, is a perfectly safe and rational one in the presence of a soft and yielding cervix. It is not however to this class of cases that I ask your attention, but rather to those cases requiring forceps when the undilated cervix is firm and unyielding.

Eclampsia and dry labors supply by far the most frequent indications for forceps under these circumstances, and in these cases dilatation of the cervix often becomes the most difficult and serious part of the delivery. It is my purpose to review the various means for dilating the cervix under such conditions, and consider the method offering the best chance of success and at the same time the one attended by the least injury.

In all such operations the evident risks are shock, infection and tears. With tears come the immediate danger of hemorrhage or the remote effects attendant upon deep cervical laceration. If we reflect for a moment upon the very slow and gradual process resulting in the obliteration of the cervix in normal labor, and then compare the means and method by which artificial dilatation accomplishes the same thing, we should hardly be surprised that even under the greatest care, lacerations occur. In normal labor the advancing membranes and head thin the tissues by gradual rearrangement of the fibers, while in artificial dilatation there is practically no softening as the os dilates, and its widening must depend upon the stretching force alone. We accomplish in one-half or one hour what nature requires ten hours to do, and it is reasonable to expect that the rigid cervix will suffer some trauma.

The means most nearly imitating nature is the use of the variously shaped hydrostatic and pneumatic bags, a class typified

1. Read before the Medical and Surgical Society of Hornell, N. Y., November 2, 1908.

by the Barnes bag. It is only, however, in cases in which time is no object that they are indicated at all. Dry labor affords the only indication in the class of cases that we are considering. Personally I have never used them under these circumstances, much preferring other means. Their use increases markedly the danger of infection, they require much attention from the physician and are more or less uncertain in their action.

The method most commonly adopted, by reason of its convenience and efficiency, is manual dilatation. One or two hands are used depending upon the strength and experience of the operator. By this plan we have the least chance of injury. The soft fingers in contact with the cervix, in themselves cause no injury and they also by their tactile sense determine the degree of tension to which the cervix may be subjected. With a soft yielding cervix there is scarcely any argument in favor of other methods of artificial dilatation. This does not mean that the method carries no dangers with it, for the most extensive cervical tear I ever saw, was the result of too great haste and ill advised force in manual dilatation. In this as in all other obstetric operations it is good judgment, experience, and a cool head that renders accident and injury least likely to occur.

In cases, however, of rigid firm cervix, manual dilatation, for the man of ordinary strength, is almost a physical impossibility and even though he succeeds, at the end of three quarters of an hour's effort, he is practically incapacitated for the effective use of forceps. The experience of all will bear testimony to this statement. In such cases we have two means at our command. One is the employment of multiple cervical incisions and delivery by forceps. This plan I have never employed as one must always have sufficient dilatation for the forceps blades, and if such dilatation has not already occurred it must be accomplished manually. It is easy to imagine that one of these incisions might readily be converted into a deep and serious laceration.

The second method is in the use of the branching dilator. The most familiar one is the Bossi instrument which seems to answer the requirements better than other models of its kind. For the past ten years many reports on its use by American and European obstetricians have appeared and the testimony has been more or less conflicting. We should remember in the use of such an instrument that the personal coefficient is an important factor and while in the hands of one man it proves itself a safe and satisfactory means, with another it may yield entirely different results.

The Bossi dilator is too well known to require description, but a more specific reference to its indications and use may not be out of place. In a general way it may be said that its indications are those in which rapid delivery is necessary in the interest of mother or child, in cases where a firm unyielding cervix renders manual dilatation difficult or impossible. Eclampsia will, therefore, most often bespeak its use. Cases of dry labor in which the woman has become exhausted and the child in danger furnish other instances. In the induction of premature labor and delivery, it finds a rare indication. I have used it myself once under such circumstances. The instrument is as applicable in cases where no dilatation has occurred, as in those where labor has proceeded to some degree.

The details in the technic in its use are important. The first essential in the preparation for artificial dilatation by any method, is proper position. Such an operation should never be attempted until the patient is in the lithotomy position upon a firm table with the hips well to the edge. The antisepsis in every detail should be most rigorous. In addition to the dilator there should be at hand suture material, needles and holder. A weighted speculum, two strong volsellum forceps and an intra-uterine douche tip complete the essentials. If possible there should be one assistant physician. The use of gloves in these cases, I think, is a disadvantage as the bare hand is much better able to appreciate the indications of too great tension and stretching. Bladder and rectum should receive proper attention. Chloroform to the surgical degree is a great aid. Work is much hampered by a resisting patient whose muscles are in a state of tension. In primiparæ it expedites matters to devote a few minutes to the stretching of the vaginal outlet, until the hand can be passed easily into the vagina. Time taken for this purpose is not lost for it facilitates the dilatation and renders the subsequent forceps-delivery easier.

The weighted speculum is then placed in position, the anterior lip of the cervix being seized by the volsellum forceps and drawn well down. The dilator is then introduced into the cervix with or without the detachable tips, depending upon the degree to which the os may already be open. As the dilation proceeds the shoulders of the tips will hold the instrument in place and the volsellum forceps may be removed. Care should be taken to see that the shoulders are well inside the uterus and not against the cervix itself.

There are several suggestions to be constantly borne in mind which will minimise the danger of laceration. In increasing the dilation the fingers should be kept on the rim of the cervix in order to gauge the tension that it is safe to produce. The screw should be released frequently in order to allow a return of circulation in the stretched tissue. The position of the instrument should be changed from time to time so that the pressure will not be at the same points continuously. It is impossible, of course, to lay down any rule as to the rate of dilatation, as all cases differ and the finger must be depended upon to indicate what each cervix will stand without laceration. It must be borne in mind in this connection that the indicator on the handle shows the dilatation under tension, and when that tension is relaxed the actual working size of the cervical ring is somewhat less.

All the criticism of the instrument is centered in its possibilities for injury and it must be conceded that carelessly used it may cause deep tears. I am inclined to believe, however, that some of the lacerations reported to be present after its use are the result of dragging the head through the cervix, and thus extending deeply by the forceps a comparatively slight tear caused by the dilator. This I know to have occurred in one of my own cases. Granting, however, that more or less laceration may occur, it seems hardly a valid reason for discarding the instrument, for in cases where the dilator finds indication the advantage for speedy delivery far overbalances a possible lacerated cervix in bringing it about.

As illustration, permit me to cite two cases:

CASE 1.—Mrs. D., 24 years old, in her first pregnancy was seized with violent convulsions about the time of her expected confinement. After the first few seizures return to consciousness did not take place but the convulsions recurred frequently. I saw her about seven hours after her first seizure. Chloroform and morphine had been used freely. She presented the usual picture of the advanced eclamptic; pulse rapid and weak; fetal heart rapid and the sounds indistinct. Immediate delivery was suggested with a view to possibly saving the child. Cervix firm and unyielding admitting a finger. Dilation with the Bossi and forceps delivery required one hour and twenty minutes. The eight pound child was born dead. The mother died four hours later. This case was a particularly difficult one to dilate and had I dilated manually, I am sure that I should have been obliged to resign the forceps to some one else.

CASE 2.—One morning Dr. Potter of Niagara Falls asked me to see sometime during the day Mrs. V., who was within three weeks of her second confinement. Despite appropriate treatment her urine contained increasing amounts of albumin and headache was beginning to be a troublesome feature. I saw the patient at two in the afternoon and learned that since I had been called her eyesight had failed completely. During my examination, a convulsion of moderate severity occurred. The cervix was undilated, thick, and unyielding. Immediate dilation and delivery required fifty minutes. Three mild convulsions occurred postpartum. Mother and child both did well. Her urine never cleared up and the findings indicated an old nephritis.

Among the seven cases in which I have used the instrument two were of dry labor in which the patients were exhausted. Rigid firm cervix in both cases. In one I might have applied forceps at once but preferred first to dilate further. In none of the cases was there any serious tear.

In conclusion the views that I have tried to express in the foregoing are as follows: hydrostatic bags have a limited field in certain cases in which time is no object. Manual dilatation is of all methods the best where the strength of the operator and a yielding cervix makes it feasible. Instrumental dilatation is recommended in cases where the cervix is firm and unyielding. When used with a proper understanding it is no more liable to cause serious injury than injudicious manual dilatation, and it conserves the strength of the operator—a feature which is sometimes of the greatest importance.

1248 Main Street.

